# Structure and Function of Snake Venom Proteins Affecting Platelet Plug Formation

**DOI:** 10.3390/toxins2010010

**Published:** 2009-12-28

**Authors:** Taei Matsui, Jiharu Hamako, Koiti Titani

**Affiliations:** 1 Department of Biology, Faculty of Medical Technology, Fujita Health University School of Health Sciences, Toyoake, Aichi 470-1192, Japan; 2 Department of Physiology, Faculty of Medical Information Technology, Fujita Health University School of Health Sciences, Toyoake, Aichi 470-1192, Japan; Email: jhamako@fujita-hu.ac.jp; 3 Division of Medical Polymer Sciences, Institute for Comprehensive Medical Sciences, Fujita Health University, Toyoake, Aichi 470-1192, Japan; Email: ktitani@crest.ocn.ne.jp

**Keywords:** disintegrin, snake venom, von Willebrand factor, platelet GPIb, thrombosis

## Abstract

Many snake venom proteins have been isolated that affect platelet plug formation by interacting either with platelet integrins, membrane glycoprotein Ib (GPIb), or plasma von Willebrand factor (VWF). Among them, disintegrins purified from various snake venoms are strong inhibitors of platelet aggregation. Botrocetin and bitiscetin derived from *Bothrops jararaca* and *Bitis arietans* venom, respectively, induce VWF-dependent platelet agglutination *in vitro*. Several GPIb-binding proteins have also been isolated from snake venoms. In this review, we focus on the structure and function of those snake venom proteins that influence platelet plug formation. These proteins are potentially useful as reagents for the sub-diagnosis of platelet disorder or von Willebrand disease, as well as for clinical and basic research of thrombosis and hemostasis.

## 1. Introduction

Platelet plug formation is an important step in hemostasis preceding the initiation of the coagulation cascade. Plasma von Willebrand factor (VWF) and its platelet membrane receptors, glycoproteins (GP) Ib and IIb/IIIa, are a prerequisite for this first event [1,2]. When subendothelial matrices such as collagens are exposed at the site of vascular damage, VWF immediately sticks to them leading to the conformational change under high shear stress and platelets start rolling on the immobilized VWF *via* its surface GPIb molecules. The continuous interaction between VWF and GPIb potentiates platelet inside-out signal transduction, leading to the conformational change of cryptic GPIIb/IIIa molecules. GPIIb/IIIa is an integrin alpha IIb, beta 3 that recognizes the Arg-Gly-Asp (RGD) sequence in fibrinogen or VWF, and these interactions induce platelet-platelet aggregation to form a primary platelet plug. The coagulation sequence is initiated on the surface of activated platelets and the resulting fibrin clot strengthens the platelet plug by trapping red blood cells into the sticky mesh-like structure.

Snake venoms contain a variety of bioactive substances that influence hemostasis, thrombosis, and coagulation of mammalian blood [3,4,5,6]. In this short review, we focus on snake venom proteins that affect platelet plug formation. These proteins have been used not only as specific reagents for the basic study of thrombosis and hemostasis, but are also expected to have clinical applications, e.g., as anti-thrombotic or diagnostic reagents [7,8,9].

## 2. Structure and Function of Disintegrins

The first disintegrin (trigramin) was discovered and purified from the venom of *Trimeresurus gramineus* by Huang *et al*. in 1987 [10] as a strong platelet aggregation inhibitor [11]. During the last two decades, more than 78 disintegrins have been reported from snake venoms. Recently, a standardized scientific classification and nomenclature for disintegrins has been proposed [12]. Disintegrins are relatively Cys-rich small polypeptides (Mr: 5~15 kDa) that potently block the binding of fibrinogen and VWF to GPIIb/IIIa complexes in ADP- or thrombin-activated platelets [13]. The inhibitory effect of disintegrin is due to its typical RGD sequence motif that competitively interacts with integrin receptors. Since disintegrin elicits a more than 1,000-fold higher inhibitory effect on platelet aggregation compared to the linear tetrapeptide Arg-Gly-Asp-Ser, a specific conformation, stabilized for example by disulfide bridges or other synergic domains, should be responsible for its higher activity.

**Figure 1:**
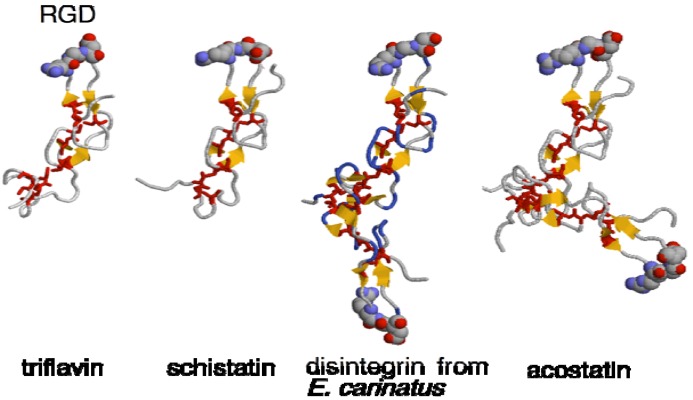
Structures of disintegrins. The 3D structures of triflavin (PDB ID; 1J2L), monomeric form of schistatin (1RMR), disintegrin from *E. carinatus* (1TEJ) and acostatin (3C05) were generated with RasMol [21]. The RGD sequence and Cys residues were shown in spacefill and sticks, respectively.

The three-dimensional (3D) structures of several disintegrins such as acostatin [14], kistrin [15], echistatin [16], triflavin (trimestatin) [17], salmosin [18], schistatin [19], disintegrin from *Echis carinatus* [20] and rhodostomin have been elucidated by NMR or X-ray crystallographic studies (Figure 1). An NMR study of linear and disulfide-looped peptides containing RGD residues has indicated that the stability of the cyclic form derives from the packing of the Arg and Asp side chains [22]. Based on the crystal structure of triflavin (trimestatin) from *Trimeresurus flavoviridis* venom, a number of turns and loops form a rigid core stabilized by six disulfide bonds. The RGD sequence is located at the tip of a hairpin loop in such a manner that the Arg and Asp side chains point in opposite directions (Figure 1). Fujii *et al*. [17] suggested that the Arg and Asp bind to the propeller domain and beta-A domain of integrin, respectively, and that the *C*-terminal region of Arg-Trp-Asn is another potential binding site with integrin receptors. Bilgrami *et al.* reported the homodimer [19] and heterodimer [20] 3D structures of the disintegrin schistatin from *E. carinatus* venom.

There is interest in applying disintegrin motifs for therapeutic purposes to prevent thrombus formation; however, the RGD motif effectively inhibits adhesive functions of other RGD-dependent integrins. There was a possibility that the RGD-containing peptide might have an adverse affect on cellular functions when administered. Since the RGD motif is not specific to GPIIb/IIIa, the specific motif for GPIIb/IIIa has been explored. Scarborough *et al*. [23] screened 62 snake venoms and found that the disintegrin, barbourin, has sole specificity to GPIIb/IIIa. Interestingly, barbourin contains a Lys-Gly-Asp (KGD) sequence within a disulfide ring instead of an RGD sequence. The KGD motif of barbourin was selected and designed as a therapeutic peptide named “eptifibatide” [24,25]. Eptifibatide elicited significant clinical benefits as an adjunctive therapy in patients undergoing selective percutaneous coronary intervention with stent implantation in the ESPRIT (Enhanced Suppression of the Platelet IIb/IIIa Receptor with Integrin Therapy) study, in patients with acute coronary syndromes in the PURSUIT (Platelet Glycoprotein IIb/IIIa in Unstable Angina: Receptor Suppression Using Integrin Therapy) trial, and in the IMPACT (Integrilin to Minimize Platelet Aggregation and Coronary Thrombosis) trial [26,27,28]. Other than eptifibatide, a recombinant barbourin-albumin fusion protein also shows an antithrombotic effect *in vivo* in animal experiments [29].

Snake venoms also contain metalloproteinases with a disintegrin domain termed ADAM [30], and ADAM family proteins have been found to take part in a variety of cellular response [31] such as sperm-egg binding (fertilin) [32], muscle fusion [33], or intracellular cleavage and activation of Notch [34]. Kaouthiagin purified from *Naja naja kaouthia* venom is an ADAM protein containing two disintegrin-like sequences [35]. Although the exact function of the disintegrin-like domain is unknown, kaouthiagin cleaves a single site of VWF resulting in the disruption of the platelet agglutination-inducing activity of VWF [36].

## 3. Structure and Function of VWF-Modulating Snake Venom Proteins

The platelet agglutination mediated by the VWF-GPIb axis never occurs under static conditions, but is inducible with the assistance of cofactors *in vitro* even under static conditions. The antibiotic ristocetin has been used as a cofactor for VWF in clinical settings to sub-diagnose von Willebrand disease or platelet disorders such as Bernard-Soulier syndrome. However, ristocetin has some limitations, such as the fact that it shows no effect on dog platelets and its working concentration is between 0.5~1.5 mg/mL; at more than 2.0 mg/mL, it precipitates fibrinogen by flocculation [37,38]. To overcome these defects, Read *et al.* screened 73 snake venoms to find a novel cofactor for VWF [39]. They found snake venoms from five species (*Bothrops alternatus*, *B. jararaca*, *B. medusa*, *B. neuwiedii*, and *Bitis arietans*) that have such cofactor activity (coagglutinin) for VWF. Two VWF-modulating venom cofactors, botrocetin and bitiscetin, have been purified and extensively studied.

Botrocetin from *B. jararaca* and bitiscetin from *B. arietans* clearly agglutinate fixed or fresh platelets in the presence of VWF irrespective of mammalian species [40]. The effective concentration for inducing VWF-dependent platelet agglutination is approximately 2 to 5 µg/mL. These cofactors are disulfide-linked heterodimers (approximately 25-27 kDa) comprised of similar α and β- subunits, each of which contains a C-type lectin-like motif, although they show neither sugar-binding nor Ca^2+^-dependent activity [41,42]. Botrocetin α- and β-subunits show a high degree of similarity to the GPIb-binding proteins from the same species, *B. jararaca* (64% and 56% for α- and β- subunits, respectively [43]), but relatively low similarity to bitiscetin (41% and 44% for α- and β- subunits, respectively) even though botrocetin has the same function as bitiscetin. Since botrocetin has a highly acidic pI (4.6), contrary to bitiscetin (9.1), they might bind to a different site on VWF; however, Ala-scanning analysis of targeted residues in the A1 domain together with crystal structural analysis clearly indicate that both proteins bind to the VWF A1 domain in very close proximity [44,45,46,47,48,49]. Obert *et al.* [50] found a second cofactor from *B. arietans* venom, bitiscetin-2, which also induces a VWF-GPIb interaction. Bitiscetin-2 binds to the A3 domain of VWF, a collagen-type III binding site, but not to the A1 domain. The partial *N*-terminal amino acid sequence of bitiscetin-2 is similar to but distinct from that of bitiscetin. They suggested that bitiscetin-2 induces a long-range conformational change of the A1 domain by acting at the A3 domain level in a way similar to that of collagen under high shear-stress conditions.

**Figure 2:**
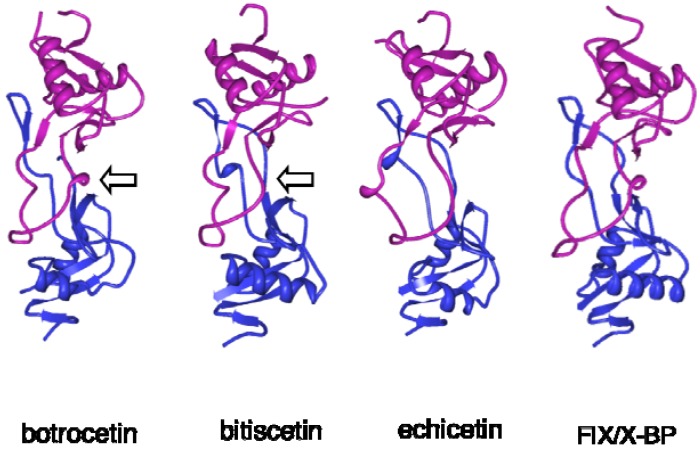
Structures of VWF- and GPIb-binding snake venom proteins. The 3D structures of botrocetin (1FVU), bitiscetin (1JWI), echicetin (3C05) and FIX/X binding protein (FIX/X-BP, 1IXX) were generated with Protein Workshop Viewer [55]. The α- and β- subunits were expressed in magenta and blue, respectively. Arrow indicates the concave region that binds to VWF A1 domain (Figure 3).

The 3D structures of botrocetin and bitiscetin were elucidated at 1.8 and 2.0 Å resolution, respectively, in 2001 (Figure 2) [45,46]. The overall 3D structures of botrocetin and bitiscetin expectedly resemble other C-type lectin-like snake venom proteins such as FIX/X-BP [51], FX-BP from *Deinagkistrodon acutus* [52], GP-Ib-binding protein (flavocetin A) from *T. flavoviridis* [53], and GPVI-binding protein (convulxin) from *Crotalus durissusterrificus* [54]. Both subunits are composed of two helices and five to eight strands, forming a globular domain with an extended loop domain (Figure 2). Each loop domain of the subunit extends to a globular domain on the opposite subunit, and they embrace each other by hydrophobic interactions in addition to an inter-subunit disulfide bridge. This bow tie-like structure provides a central concave area hinged by both subunits, serving as a ligand binding site.

**Figure 3:**
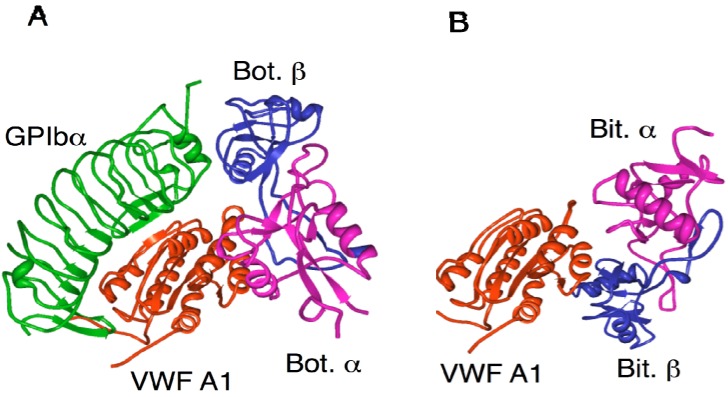
Complex structures of GPIbα-VWF A1- botrocetin and VWF A1-bitscetin. A: The 3D structures of botrocetin complexed with GPIbα (green) and VWF A1 domain (orange) [44]. Botrocetin and subunitis were expressed as magenta (Bot,α) and blue (Bot,β), respectively. B: The 3D structures of bitiscetin complexed with VWF A1 domain (orange) [48]. Bitiscetin α and β subunits were expressed as magenta (Bit.α) and blue (Bit,β), respectively. Ribbon structures were drawn with Protein Workshop Viewer [55] using PDB file of 1U0N (A) and 1UEX (B).

Botrocetin binds to VWF A1 domain *via* its concave region with comparable contributions from the α- (43%) and β- (57%) subunits. The botrocetin and bitiscetin binding sites on VWF overlap at the A1 domain, but the direction of the long-axis of bitiscetin is almost perpendicular to that of botrocetin (Figure 3). It seems likely that the β-subunit of botrocetin and the α-subunit of bitiscetin have a crucial role in interaction with GPIb. The *K_d_* values for botrocetin and bitiscetin binding to the VWF A1 domain are estimated to be 12nM [56] and 2 nM [48], respectively.

Botrocetin and bitiscetin appear to modulate VWF susceptible to GPIb by invoking an allosteric structural change on the GPIb-binding domain of VWF. However, contrary to expectation, crystal structures of the A1 domain complexed with botrocetin and bitiscetin have indicated no significant conformational changes in the GPIb binding site of the A1 domain before or after binding with botrocetin or bitiscetin [48,57]. The importance of the anionic region of the GPIb domain for botrocetin-induced VWF binding suggests that it plays an essential role in the high affinity binding of GPIb to the botrocetin-A1 complex even though botrocetin does not bind to GPIb by itself. Several important residues on GPIb for botrocetin-induced platelet agglutination have been described [58]. Bitiscetin- or botrocetin-induced binding of GPIb to VWF may depend on electrostatic interactions between the anionic region of GPIb (sulfated Tyr residues) and a complementary electropositive spot contributed by the venom protein in the VWF A1-bitiscetin or VWF-botrocetin complex. It is conceivable that bitiscetin or botrocetin complexed with the A1 domain provide a supplemental platform suited for GPIb rather than inducing an allosteric conformational change on the A1 domain (Figure 3). This new hypothesis is in good agreement with results showing that GPIb without the sulfated Tyr residues fails to bind VWF in the presence of botrocetin [59]. Fukuda *et al*. [44] have recently found that botrocetin binds to and subsequently slides on the A1 surface to form a new interface with enhanced affinity to GPIb.

## 4. Structure and Function of GPIb-binding Snake Venom Proteins

GPIb-binding proteins from snake venoms are categorized into two groups: GPIb-agonists and GPIb-antagonists. In addition to botrocetin, both groups have amino acid sequences highly similar to C-type lectin-like structures.

The first GPIb-agonist (alboaggregin-B) was purified from *Trimeresurus albolabris* venom by Peng *et al.* in 1991 [60]. Alboaggregins (A, B, and C), having highly similar primary sequences, bind to GPIb (and GPVI in the case of alboaggregin A) and induce platelet agglutination or aggregation [61]. They also inhibit VWF mediated platelet agglutination, suggesting that their binding sites overlap or are proximal to the VWF binding site on GPIb. This inhibitory effect strongly resembles GPIb-antagonists. The same activity has been found in mamushigin from *Agkistrodon halys blomhoffii* [62], bilinexin from *A. bilineatus* [63], agglucetin from *A. acutus* [64], agkicetin-C from *D. acutus* [65], mucetin (TMVA) from Chinese habu *T. mucrosquamatus* [66], and mucrocetin from Taiwan habu *T. mucrosquamatus* [67] venoms. Several GPIb-agonists induce only platelet agglutination *in vitro*, whilesome agonists further activate platelets leading to GPIIb/IIIa-dependent aggregation.

Alboaggregin A and agglucetin are tetrameric forms composed of disulfide-linked subunits [68]. Although the dimeric form is sufficient to induce platelet agglutination, the tetramer significantly enhances platelet aggregation and the release reaction. Agglucetin agglutinates formalin-fixed platelets in a GPIb-dependent manner, but also activates surface exposure of GPIIb/IIIa of intact platelets without significant elevation of intracellular Ca^2+^ mobilization and thromboxane B formation [64,68]. Mucetin also has several multimeric forms, including (αβ)_2_ and (αβ)_4_, and activates platelets *via* GPIb. The platelet activation process by the mucetin-GPIb interaction is not clear, but Lu *et al.* [66] found that signaling by mucetin involves rapid Tyr phosphorylation of Syk, Src, LAT, and PLC 2, with translocation of GPIb and the Fc receptor g-chain to the cytoskeleton [69].

**Figure 4:**
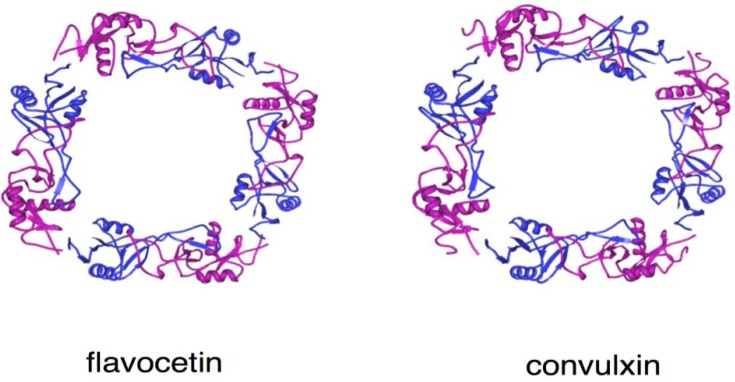
Structures of flavocetin and convulxin. The 3D structures of flavocetin-A (1C3A) and convulxin (1UMR) were drawn with Protein Workshop Viewer [55]. The α and β- subunits were expressed in magenta and blue, respectively. They forms cyclic tetramer (αβ)_4_ composed of disulfide-linked four αβ-heterodimers.

The other GPIb-binding group, the GPIb-antagonists, also specifically binds to GPIb but do not induce platelet agglutination. They competitively block binding of VWF or alboaggregin to GPIb, resulting in the inhibition of platelet agglutination. Echicetin, a disulfide-linked heterodimer of two subunits, purified from *E. carinatus* was first described as a GPIb-antagonist by Peng *et al.* in 1993 [70]. GPIb-antagonists have been purified from the venom of many snake species, including flavocetin-A and -B from *T. flavoviridis* [71], tokaracetin from *T. tokarensis* [72], jararaca GPIb-BP (yoshitobin) from *B. jararaca* [73], dabocetin from *Daboia* russellii *siamensis* [74], and agkicetin from *D. acutus* [65].

The 3D structures of flavocetin-A [75], echicetin [76], and agkicetin [77] have been elucidated (Figure 2 and Figure 4). Overall, they are similar to those of VWF-modulating proteins and GPIb-agonists, since they all belong to the C-type lectin-like protein family. Flavocetin-A is a high-molecular mass protein (149 kDa) with a unique 3D structure: a cyclic tetramer (αβ)_4_ composed of four disulfide-linked αβ-heterodimers (Figure 4) [75]. The tetramerization is mediated by an interchain disulfide bridge between Cys residues at the C-terminus of the α-subunit and the *N*-terminus of the β-subunit in the neighboring αβ-heterodimer, in a manner of a “head-to-tail” interaction. This configuration is also found in the platelet activator convulxin, purified from *C.durissusterrificus* venom, which binds to GPVI (Figure 4) [54]. Although flavocetin-A shows an inhibitory effect on platelet agglutination, it induces small platelet aggregate formation by itself through partial activation of platelets due to its multivalent GPIb binding site [78], indicating that flavocetin-A is bifunctional.

Although the 3D structure of GPIb and GPIb-binding protein complex has not been elucidated, the binding site of GPIb on GPIb-binding proteins has been examined. The biological activity of echicetin is present in the β-subunit but not α-subunit, as seen from experiments using reduced and alkylated subunits [70,79]. Two hydrophilic patches in the β-subunit of flavocetin-A are considered to be the GPIb binding site [75]. Agkicetin-C has been found to bind the region between 201 and 282 of GPIbα [65].

## 5. Perspectives

Snake venoms comprise a natural library of valuable bioactive substances for hemostasis and thrombosis. The snake venom cofactors described here are useful for clinical evaluation or sub-diagnosis of bleeding disorders as well as for basic investigation into the molecular mechanisms of platelet plug formation induced by VWF and platelets. However, it has become quite difficult to obtain crude venom samples from foreign countries after the adoption of the Washington Convention, which controls the international trade of endangered species [80]. Studies of snake venoms at the protein level might be limited to certain countries of Asia, Africa, and the Americas, where a variety of snakes range. Thus, patience is required to protect and maintain natural scarce animals so that recombinant studies at the genetic level might lead to numerous benefits.

Disintegrins have shed light on drug design of anti-platelet plugs and elicited efficient anti-hemostatic effects in several therapeutic trials [81]. More diverse clinical trials should be focused toward a variety of thrombotic diseases. Formulation of the tablet form of disintegrin, which is more stable, may be feasible through coupling to digestive proteins.

For VWF cofactor proteins, evaluation of VWF activity using these proteins should be superior to ristocetin in sensitivity and accuracy. Recombinant cofactor proteins purified to homogeneity are expected to be used as a standard assay reagent instead of ristocetin in the future. A goal is to find a novel VWF-binding protein that blocks the interaction between GPIb and VWF, making it more preferable for controlling platelet plug formation as it blocks the GPIb binding activity of VWF without inducing signal transduction in platelets.

GPIb antagonists inhibit platelet agglutination *in vitro*; however, echicetin or agkicetin induce thrombocytopenia when injected in rats, indicating that unknown interactions leading to platelet aggregation are invoked *in vivo* by administration of these antagonists. In physiological circulating blood, shear-stress might also give rise to an effective signal on platelets complexed with GPIb-binding proteins. Further, immunological response would exclude snake venom-derived materials when it was administered long-term, since there was no similar protein in human to the C-type lectin-like snake venom proteins. These results suggest that GPIb-antagonists are not clinically applicable at present, but if the key domain for eliciting GPIb binding could be straitened like a peptide aptamer [82], anti-platelet plug drugs could result. Furthermore, both GPIb-antagonists and -agonists are potential reagents for investigating the platelet activation pathway originating from GPIb [83,84].

The snake is a symbol of medical knowledge from ancient times. Fittingly, the continuing study of snake venom components is bringing to light useful knowledge of thrombosis and hemostasis.
